# RASGRP2 Suppresses Apoptosis via Inhibition of ROS Production in Vascular Endothelial Cells

**DOI:** 10.1155/2019/4639165

**Published:** 2019-01-01

**Authors:** Takuma Sato, Jun-ichi Takino, Kentaro Nagamine, Kazuto Nishio, Takamitsu Hori

**Affiliations:** ^1^Laboratory of Biochemistry, Hiroshima International University, Hiroshima, Japan; ^2^Department of Genome Biology, Kindai University Faculty of Medicine, Osaka, Japan

## Abstract

We have identified* ras guanyl releasing protein 2* (*rasgrp2*) as a blood vessel related gene from Xenopus embryo. In addition, we reported that RASGRP2 is also expressed in human umbilical vein endothelial cells (HUVEC). It is known that RASGRP2 activates Ras-related protein 1 (Rap1). However, the function of RASGRP2 in human vascular endothelium remains unknown. Therefore, we performed functional analysis of RASGRP2 using immortalized HUVEC (TERT HUVEC). We established a stable RASGRP2 overexpressing cell line (TERT HUVEC R) and mock cell line (mock). Furthermore, we compared the activity of Rap1 and the generation of intracellular reactive oxygen species (ROS), which is related to cell death, in both cell lines. Significant increase in Rap1 activity was observed in the TERT HUVEC R compared to the mock. Furthermore, apoptosis by tumor necrosis factor-*α* (TNF-*α*) stimulation was significantly more reduced in the TERT HUVEC R than in the mock. In the mock, apoptosis induced by TNF-*α* stimulation was decreased by pretreatment with diphenyleneiodonium (DPI), which is an inhibitor of NADPH oxidase (NOX). However, in the TERT HUVEC R, apoptosis induced by TNF-*α* stimulation was not reduced after pretreatment of DPI. Furthermore, there was no reduction in ROS production in the TERT HUVEC R after DPI pretreatment. In addition, the difference in the degree of apoptosis induced by TNF-*α* stimulation in both cell lines was consistent with the difference in ROS production in the cell lines. From these results, it was suggested that RASGRP2 activates Rap1 and the activated Rap1 suppresses apoptosis via NOX inhibition.

## 1. Introduction

Vascular endothelial cells are exposed to various cytokines such as interleukins and growth factors under inflammation and tumor microenvironment [1, 2]. Among them, tumor necrosis factor-*α* (TNF-*α*) is one of the most important inflammatory cytokines. TNF-*α* is produced by many cell types, including macrophages, lymphocytes, and fibroblasts in response to inflammation, infection, and other stresses [3]. In addition, TNF-*α* has been reported to be involved in the generation of reactive oxygen species (ROS), hyperpermeability, and apoptosis in vascular endothelial cells [4–6].

We have identified Xenopus RAS guanyl releasing protein 2 (RASGRP2) as a blood vessel related gene from Xenopus embryo [7–9]. Furthermore, we reported that Xenopus RASGRP2 has highly homology to human RASGRP2 [8]. RASGRP2 is well known as guanine nucleotide exchange factor (GEF) [9]. GEF stimulates guanosine triphosphate (GTP) loading of small G proteins and are competed by GTPase activating proteins which catalyzes GTP hydrolysis [10]. In addition to GEF, RASGRP2 is a protein with EF-hand, CDC25 domain, Ras exchange motif, and diacylglycerol binding C1 domain [9]. The C1 domain constitutes the recognition module for diacylglycerol in RASGRP [11]. RASGRP family is known to consist of four members [12]. Among RASGRP family members, the C1 domain of RASGRP2 is characterized by a weak affinity for diacylglycerol [12]. Furthermore, it has also been reported that amino-terminal region of RASGRP2 can bind to polymerized actin* in vitro* [10].

RASGRP2 has been reported to have an important role in platelets and leukocyte [13–15]. For example, RASGRP2 activates platelets by activating in integrins and contributes to the formation of thrombi [13]. In addition, RASGRP2 is involved in the role of neutrophil chemotaxis* in vitro* and the mobilization of neutrophils into the inflamed peritoneal cavity* in vivo *[14, 15]. In T cells, it has been reported that RASGRP2 enhances the adhesion ability of lymphocyte function-associated antigen-1 and contributes to the interaction with intercellular adhesion molecule-1 [16]. In addition, there are several reports that RASGRP2 is involved in leukemia [17, 18]. RASGRP2 has been identified as the proto-oncogene in acute myelogenous leukemia [17]. Furthermore, it is suggested that RASGRP2 is increased in expression in trisomy 12-associated chronic lymphocytic leukemia and contribute to the enhanced integrin signaling associated with drug resistance [18].

Rap1 has been reported to be activated not only by RASGRP2 but also by GEF such as EPAC and C3G and furthermore downregulated by Rap1GAP1 and SPA-1 [19]. EPAC in HUVEC is regulated by cAMP to activate the Rap1, and it has been reported to enhance the vascular endothelial barrier function [20]. Furthermore, in vascular endothelial cells C3G is reported to recover the barrier function by activating Rap1 in enhancement of permeability by thrombin [21].

We recently reported that RASGRP2 activates Rap1 in ECV304 of the human bladder epithelial cancer cell line [9]. In addition, we previously reported that RASGRP2 is expressed in human umbilical artery endothelial cells and human umbilical vein endothelial cells (HUVEC) [11]. However, the role of RASGRP2 in human vascular endothelial cells exposed to TNF-*α* has not been elucidated.

In this study, the effect of RASGRP2 in presence of TNF-*α* stimulation was analyzed using TERT HUVEC.

## 2. Materials and Methods

### 2.1. Cell Culture and Transfection

Cells were maintained in the medium using Endothelial Cell Growth Medium (PromoCell, Heidelberg, Germany). pEB Multi-Hyg (Wako Pure Chemicals, Osaka, Japan) was used as vector to prepare TERT HUVEC R and mock cell lines. The DNA fragment of* rasgrp2* was isolated by the method previously described [9]. ViaFect™ Transfection Reagent (Promega, Madison, WI, USA) was used as the transfection reagent. Cells at the concentration of 0.5 × 10^5^ cells/mL were seeded in a 24-well plate, grown overnight, and transfected. Transfected cells were purified with 50 *μ*g/mL Hygromycin B solution (Nacalai Tesque Inc, Tokyo, Japan).

### 2.2. Preparation of Cell Lysate and Western Blot Analysis

Cells were lysed using IP Lysis Buffer (Thermo, Waltham, MA, USA) and centrifuged at 12000 g, and the supernatant was recovered. Then, cell lysates were dissolved in LDS sample buffer (Invitrogen, Carlsbad, CA) containing 10% sample reducing agent (Invitrogen), boiled for 10 min at 70°C, separated by SDS-PAGE, and then electrotransferred onto polyvinylidene difluoride (PVDF) membranes (Millipore Bedford, MA, USA). Membranes were blocked for 30 min using the PVDF blocking agent for Can Get Signal® (Toyobo, Tokyo, Japan). After washing with PBS containing 0.05% Tween 20 (PBS-T), the membranes were incubated with rabbit anti-RASGRP2 antibody (GeneTex, Irvine, California, USA), mouse anti-*β*-actin antibody (Santa Cruz, USA), rabbit anti-NOX4 antibody (GeneTex), or rabbit anti-Rap1 antibody (Santa Cruz) in Can Get Signal® Solution 1 (Toyobo) for 1 h. Subsequently, the membranes were washed thrice with PBS-T and incubated with anti-rabbit IgG antibody (GeneTex) or anti-mouse IgG antibody (Dako-cytomation, USA) in Can Get Signal® Solution 2 (Toyobo) for 1 h. After five additional washes with PBS-T, immunoreactive proteins were detected using ECL Prime Western Blotting Detection Reagents and Amersham Hyperfilm™ ECL (GE HealthCare, NJ, USA).

### 2.3. Rap1 Activity Assay

Rap1 activity was examined using RalGDS-RBD Agarose Beads (Cell Biolabs, San Diego, CA). Briefly, cell lysates (100 *μ*g) and RalGDS-RBD Agarose Beads (20 *μ*L) were incubated for 1 h at 4°C with gentle rotation. The beads were then washed thrice with excess lysis buffer, and then the bound proteins were eluted in LDS sample buffer.

### 2.4. Measurement of Cell Viability by TNF-*α* Stimulation

One hundred microliters of cells from both TERT HUVEC R cells and mock cells were seeded into each well at a density of 2.0 × 10^5^ cells/mL in a 96-well plate and treated with 20 ng/mL TNF-*α* (PeproTech, Rocky Hill, NJ, USA) for either 24 h or 48 h. As a pretreatment step, the cells were treated with either 5 mM N-acetyl cysteine (NAC) (Wako), 20 *μ*M diphenyleneiodonium (DPI), or 30 *μ*M apocynin (Cayman Chemical, MI, USA) for 2 h. After treatment with TNF-*α*, 10 *μ*L/well WST-8 (Dojindo Laboratory, Kumamoto, Japan) was added. Absorbance was measured after 2 h (450 nm and 650 nm), and cell viability was evaluated.

### 2.5. Measurement of Apoptosis

As with WST-8, cells of the TERT HUVEC R cells and mock cells were seeded in 96-well plates at a density of 2.0 × 10^5^ cells / mL in each well and treated with 20 ng/ml TNF-*α* for 24 h. Similar to pretreatment, cells were treated with NAC and DPI for 2 h each. After treatment with TNF-*α*, cells were treated with 1 *μ*M NucView 488 (Biotium Inc., Cambridge, UK) for 30 min and the medium was then exchanged. After obtaining the fluorescence image, apoptosis was evaluated using ImageJ, and the fluorescence area was indicated by average of randomly three selected fields.

### 2.6. Measurement of ROS

As with the measurement of apoptosis, 100 *μ*L of cells from both the TERT HUVEC R cells and mock cells were seeded in each well of a 96-well plate at a density of 2.0 × 10^5^ cells/mL. Then, the cells were treated with 20 ng/mL TNF-*α* for 4 h. Similar to pretreatment, cells were treated with 5 mM NAC and 20 *μ*M DPI for 2 h each. After treatment with TNF-*α*, the cells were treated with 5 *μ*M CellROX® Green (Thermo Fisher Scientific, Massachusetts, USA) for 1 h and then the medium was exchanged. After acquiring the fluorescence image, ROS was evaluated using ImageJ, and the fluorescence area was indicated by average of randomly three selected fields.

### 2.7. Knockdown of Rap1 by Small Interfering RNA

Ninety nanomolars of Rap1 siRNA (SASI_Hs01_00040403) or negative control siRNA (SIC-001) (SIGMA Aldrich, St. Louis, MO, USA) were transfected into cell with according to the manufacturer's instructions. Briefly, cells were seeded at 2.0 × 10^5^ cells/mL and, after 6 h, medium was changed to 10% FCS Endothelial Cell Growth Medium 2 (PromoCell) without heparin. Then, a mixture of MISSION® siRNA Transfection Reagent (SIGMA Aldrich) and siRNA was preincubated for 10 min and transfection was performed.

### 2.8. Statistical Analysis

All experiments were repeated twice at least. Each experiment yielded essentially similar results. Data is expressed as the mean ± standard deviation (SD). The significance of differences between group means was determined using a one-way analysis of variance. P < 0.01 was defined as significant result.

## 3. Results 

### 3.1. Effect of RASGRP2 Overexpression on Rap1 Activity and NOX Expression

**Figure 1:**
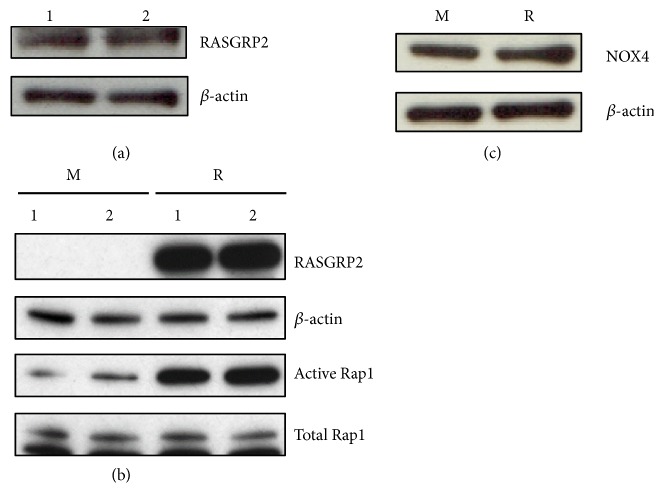
Effect of RASGRP2 overexpression on Rap1 activation and NOX4 expression. (a) Expression of endogenous RASGRP2 protein with overexposure using X-ray film. 1: HUVEC, 2: TERT HUVEC. (b) Activated Rap1 in TERT HUVEC was recovered using RalGDS-RBD Agarose Beads and validated by western blot. M: mock cells; R: TERT HUVEC R cells. 1 and 2: each of two single cell clones obtained by establishment. (c) Expression of NOX4 protein in TERT HUVEC.

The expression of RASGRP2 in TERT HUVEC was slight, and its expression was equivalent to HUVEC (Figure 1(a)). To investigate the effect of RASGRP2 in endothelial cells, we established TERT HUVEC (TERT HUVEC R) cells stably transfected with RASGRP2. The establishment of stable overexpression line was confirmed using western blotting. As a result, each of two single cell clones of mock cells and TERT HUVEC R cells was obtained (Figure 1(b)). In addition, we analyzed Rap1 activation by RASGRP2 using a pull-down method based on RalGDS-RBD. The result showed that Rap1 activation was substantially increased in the TERT HUVEC R cells than in the mock cells (Figure 1(b)). In subsequent experiments, clones of TERT HUVEC R1 cells and mock2 cells were used.

Next, since TNF-*α* produces ROS via NOX, we confirmed the effect of NOX expression by RASGRP2. As a result, it was shown that NOX4 which is prominent expressed in HUVEC has no effect by RASGRP2 (Figure 1(c)).

### 3.2. Effect on Cell Viability by TNF-*α* Stimulation

**Figure 2:**
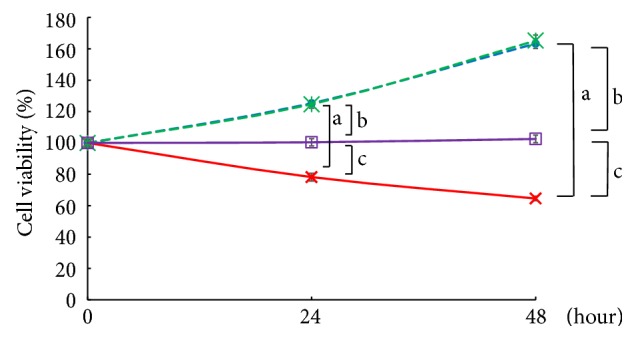
Cell viability by TNF-*α* stimulation. Cells were treated with 20 ng/mL TNF-*α* for either 24 h or 48 h. Cell viability at 0 h was taken as 100%. Blue circle: Mock untreated; red cross: Mock TNF-*α*; green asterisk: TERT HUVEC R untreated; purple square: TERT HUVEC R TNF-*α*; n=3; a = P<0.01; Mock untreated versus Mock TNF-*α*; b = P<0.01, TERT HUVEC R untreated versus TERT HUVEC R TNF-*α*; c = P<0.01, Mock TNF-*α* versus TERT HUVEC R TNF-*α*.

To investigate the effect of cell viability by TNF-*α* stimulation, we measured with WST-8. In both TERT HUVEC R and mock cell lines, cell viability was significantly decreased by TNF-*α* stimulation compared to untreated. However, the decrease was slight in TERT HUVEC R cells compared to mock cells (Figure 2). These results were similar for each other clones (data not shown).

**Figure 3:**
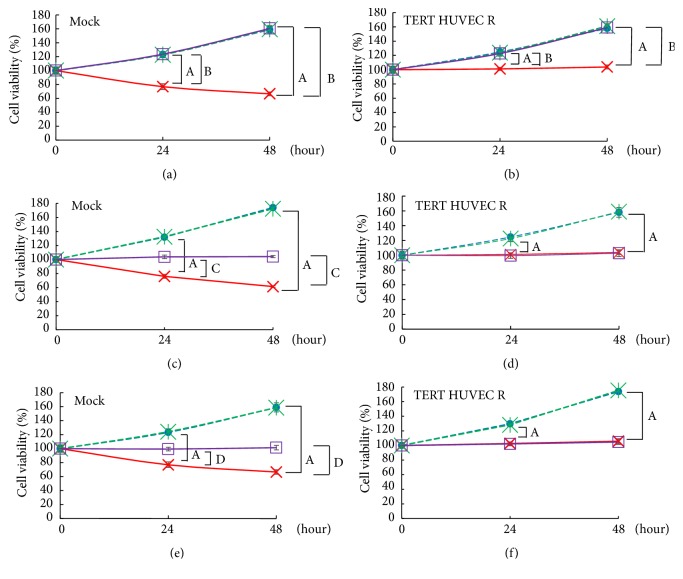
Effect of ROS inhibitors on TNF-*α* stimulation. Cells were treated with 20 ng/mL TNF-*α* for either 24 h or 48 h. Cell viability at 0 h was taken as 100%. (a) and (b) represent the cell viability of the mock cells and TERT HUVEC R cells pretreated with 5 mM NAC, respectively. Blue circle: untreated; red cross: TNF-*α*; green asterisk: NAC; purple square: TNF-*α* + NAC. (c) and (d) represent the cell viability of mock cells and TERT HUVEC R cells pretreated with 20 *μ*M DPI, respectively. Blue circle: untreated; red cross: TNF-*α*; green asterisk: DPI; purple square: TNF-*α* + DPI. (e) and (f) represent the cell viability of mock cells and TERT HUVEC R cells pretreated with 30 *μ*M apocynin, respectively. Blue circle: untreated; red cross: TNF-*α*; green asterisk: apocynin; purple square: TNF-*α* + apocynin. n=3; A = P<0.01, untreated versus TNF-*α*; B = P<0.01, TNF-*α* versus TNF-*α* + NAC; C = P<0.01, TNF-*α* versus TNF-*α* + DPI; D = P<0.01, TNF-*α* versus TNF-*α* + apocynin.

Next, in order to confirm whether reduction of cell viability by TNF-*α* stimulation is due to ROS, we investigated using NAC for ROS scavenger and DPI and apocynin for NOX inhibitor. In both cell lines, the decrease in cell viability due to TNF-*α* stimulation was completely recovered by NAC pretreatment (Figures 3(a) and 3(b)). On the other hand, in the pretreatment of DPI, it partially recovered in mock cells but did not recover in TERT HUVEC R cells (Figures 3(c) and 3(d)). Furthermore, partial recovery of mock cells in cell viability was equivalent to TERT HUVEC R cells by TNF-*α* stimulation with or without DPI pretreatment (Figures 3(c) and 3(d)). In addition, apocynin pretreatment resulted in equivalent to pretreatment of DPI (Figures 3(e) and 3(f)). From these results, it was shown that RASGRP2 in TERT HUVEC suppresses decrease of cell viability by inhibiting NOX.

### 3.3. RASGRP2 Contributes to the Suppression of TNF-*α*-Induced Apoptosis

**Figure 4:**
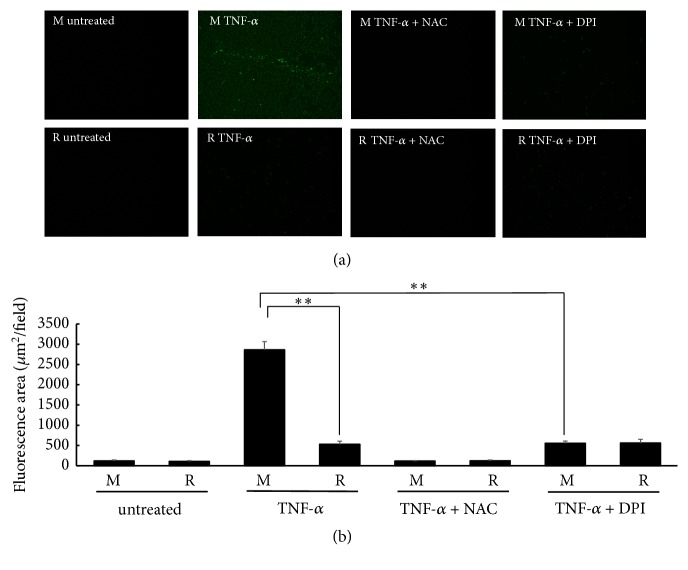
Apoptosis by TNF-*α* stimulation. Each cell line was treated with 20 ng/mL TNF-*α* for 24 h and stained using NucView 488. (a) Cells were photographed with fluorescence microscopy (40× magnification). (b) A fluorescent image was taken and digitized using ImageJ. M: mock cells, R: TERT HUVEC R cells. n=3; *∗∗*p < 0.01.

We investigated whether RASGRP2 suppress TNF-*α*-induced apoptosis using NucView 488 which can detect caspase activity. Considerable fluorescence was observed in mock cells by TNF-*α* stimulation, and it was shown that apoptosis was induced (Figure 4(a)).  Figure 4(b) is a result of digitized the fluorescence of the image. In both cell lines, apoptosis was significantly increased by TNF-*α* stimulation as compared with untreated. However, the increase in apoptosis by TNF-*α* stimulation was slight in TERT HUVEC R cells compared to mock cells. On the other hand, apoptosis induced by TNF-*α* stimulation with NAC pretreatment was equivalent to untreated cells in both cell lines. Therefore, apoptosis was completely suppressed by NAC pretreatment. Apoptosis induced by TNF-*α* stimulation with DPI pretreatment was significantly decreased in mock cells, but not in TERT HUVEC R cells. Furthermore, both cell lines did not decrease to the same level as untreated. Thus, it was shown that RASGRP2 is involved in the suppression of apoptosis via NOX inhibition.

### 3.4. RASGRP2 Suppresses TNF-*α*-Induced ROS Production

**Figure 5:**
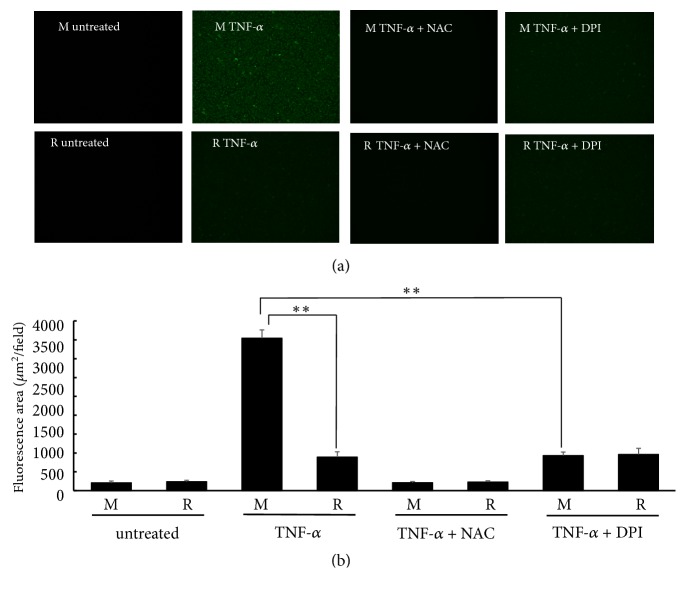
Change in intracellular ROS amount by TNF-*α* stimulation. Each cell line was treated with 20 ng/mL TNF-*α* for 4 h and stained using CellROX® Green. (a) Cells were photographed with fluorescence microscopy (40× magnification). (b) A fluorescent images were taken and digitized using ImageJ. M: mock cells; R: TERT HUVEC R cells. n=3; *∗∗*p < 0.01.

From the results of cell viability and apoptosis experiments, it was shown that RASGRP2 influences the function of NOX. Therefore, we evaluated ROS generation by TNF-*α* using CellROX® Green. Considerable fluorescence was observed in mock cells by TNF-*α* stimulation, and it was shown that ROS was generated (Figure 5(a)).  Figure 5(b) is a result of digitized the fluorescence of the image. In both cell lines, ROS levels were significantly increased by TNF-*α* stimulation compared to untreated. However, the increase in ROS caused by TNF-*α* was slight degree in TERT HUVEC R cells compared to mock cells. This increase in ROS was consistent with an increase in apoptosis by TNF-*α* stimulation. On the other hand, pretreatment of NAC in both cell lines reduced the produced ROS by TNF-*α* stimulation to the same level as untreated. Pretreatment of DPI in mock cells reduced the produced ROS by TNF-*α* stimulation, but not in TERT HUVEC R cells. In addition, pretreatment of DPI in both cell lines showed no decrease in ROS to the same level as untreated. Therefore, it was shown that RASGRP2 is involved in the suppression of ROS via NOX inhibition, but ROS generation without NOX was not suppressed.

### 3.5. NOX Inhibition by RASGRP2 via Rap1

**Figure 6:**
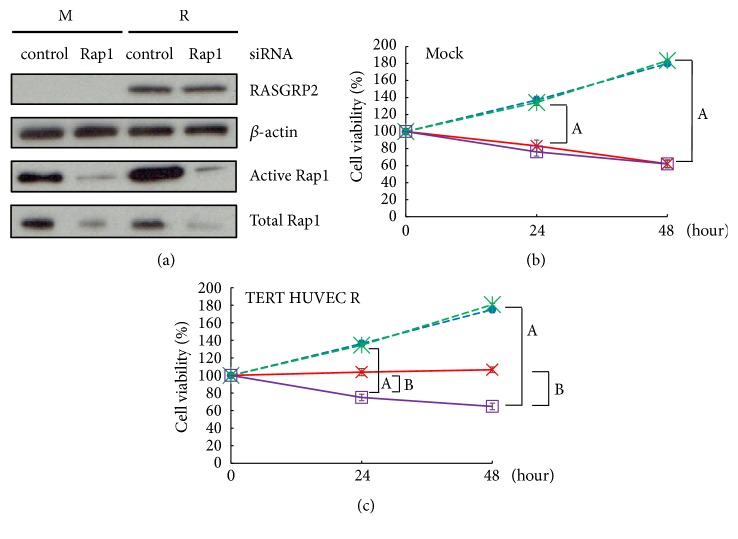
Effect of Rap1 siRNA on TNF-*α* stimulation. (a) Activated Rap1 in TERT HUVEC under Rap1 siRNA conditions was recovered using RalGDS-RBD agarose beads and validated by western blot. M: mock cells; R: TERT HUVEC R cells. (b) and (c) represent the cell viability of mock cells and TERT HUVEC R cells in TNF-*α* stimulation under condition Rap1 knocked down. Blue circle: control siRNA; red cross: control siRNA + TNF-*α*; green asterisk: Rap1 siRNA; purple square: Rap1 siRNA + TNF-*α*; n=3; A = P<0.01, Rap1 siRNA versus Rap1 siRNA + TNF-*α*; B = P<0.01, control siRNA + TNF-*α* versus Rap1 siRNA + TNF-*α*.

We performed knockdown by transfection of siRNA to investigate whether Rap1 is involved in the suppression of TNF-*α*-induced apoptosis by RASGRP2. Indeed, siRNA suppressed the expression of Rap1 in both cell lines and further reduced the active Rap1 (Figure 6(a)). Next, we investigated the effect on cell viability by TNF-*α* stimulation under knockout condition with Rap1 siRNA. As a result, in mock cells, cell viability was decreased by TNF-*α* stimulation with or without Rap1 siRNA (Figure 6(b)). On the other hand, in TERT HUVEC R cells, when TNF-*α* stimulation was performed, Rap1 siRNA significantly decreased cell viability as compared with control siRNA (Figure 6(c)). Furthermore, the significant decrease in cell viability of this Rap1 siRNA was equivalent to mock cells by TNF-*α* stimulation with or without Rap1 siRNA (Figures 6(b) and 6(c)). From these results, it was shown that RASGRP2 inhibits the TNF-*α* signaling pathway by activating Rap1.

## 4. Discussion

**Figure 7:**
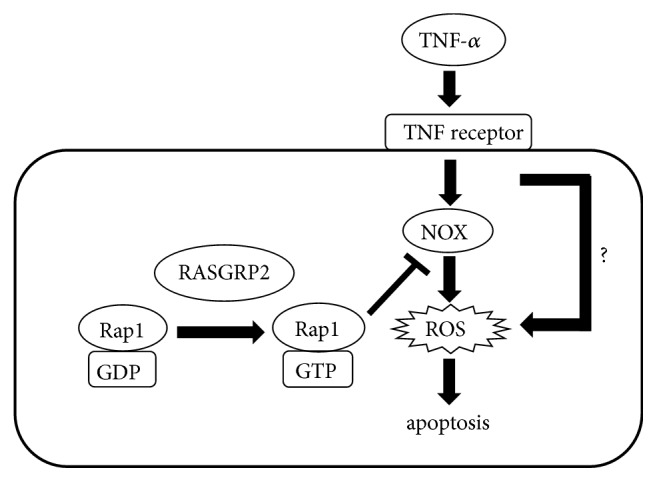
Proposed model of signaling pathway involving RASGRP2 in vascular endothelial cells.

In this study, it was shown that RASGRP2 activated Rap1 in TERT HUVEC cell line. In both cell lines, TNF-*α* did not affect the activity of Rap1 (data not shown). In addition, cell viability was decreased by TNF-*α* and its decrease was due to apoptosis (Figures 2 and 3). Furthermore, it was shown that RASGRP2 expression inhibited NOX-mediated ROS production stimulated by TNF-*α* and also suppressed apoptosis (Figure 7). However, generation of ROS by TNF-*α* stimulation was not completely suppressed in the TERT HUVEC R cells. Thus, it was shown that RASGRP2 did not suppress ROS generated via any mechanism other than NOX mediated pathway. NOX family consists of five NOX members (NOX1-5) and two DUOX members [22]. HUVEC expresses NOX2 and NOX4; however, NOX4 expression is more prominent [23]. Moreover, it has also been reported that NOX2 and NOX4 cause apoptosis by generating ROS due to TNF-*α* stimulation [22, 24]. Thus, it was suggested that RASGRP2 inhibits apoptosis by suppressing ROS generated from NOX2 and NOX4. However, the expression of NOX4 in both cell lines was no difference (Figure 1(d)). This result indicated that RASGRP2 affects the activity without changing the expression of NOX4. It has been reported that active Rap1 inhibits the activity of NOX, thereby suppressing the generation of ROS [25]. Indeed, suppression of active Rap1 by siRNA counteracted the effect of RASGRP2 (Figure 6(c)). Therefore, it was suggested that RASGRP2 contributes in maintenance of vascular homeostasis in vascular endothelial cells.

On the contrary, it has already been observed in vascular endothelial cells, using siRNA transfection and overexpression analyses, that NOX4 plays an important role in angiogenesis including endothelial cell proliferation, migration and lumen formation [26]. No significant difference was observed in the cell viability of the TERT HUVEC R cells and the mock cells treated with WST-8 (Figures 3(a) and 3(b)). In addition, no difference was found in lumen formation ability of the TERT HUVEC R cells and the mock cells using Matrigel (data not shown). Thus, it was suggested that RASGRP2 is not involved in the suppression of angiogenesis.

It has been reported that RASGRP2 is phosphorylated by protein kinase A and protein kinase C [12, 27]. Regulation of the activity of the RASGRP family has been reported to be involved in phosphorylation [12]. Indeed, it has been reported that the ability of RASGRP2 to activate Rap1 decreases when serine-587 is phosphorylated by protein kinase A [27]. Therefore, in order to elucidate the function of RASGRP2, it is also important to analyze not only its expression levels but also the signaling pathway involved in phosphorylation of RASGRP2.

Finally, in this study only TNF-*α* was used as an apoptosis-inducing agent in TERT HUVEC cells. However, it has been reported that TGF-*β* also causes apoptosis by ROS generation via NOX4 [28]. Therefore, RASGRP2 is also expected to suppress apoptosis induced by TGF-*β*.

## 5. Conclusions

In conclusion, RASGRP2 was shown to activate Rap1. Furthermore, it was suggested that activated Rap1 suppresses ROS produced via NOX by TNF-*α* stimulation and also suppresses apoptosis. In order to further elucidate the function of RASGRP2 in vascular endothelial cells, more research is needed in the future.

## Data Availability

The data used to support the findings of this study are available from the corresponding author upon request.

## References

[1] Hanahan D., Coussens L. M. (2012). Accessories to the crime: functions of cells recruited to the tumor microenvironment. *Cancer Cell*.

[2] Zhang C. (2008). The role of inflammatory cytokines in endothelial dysfunction. *Basic Research in Cardiology*.

[3] Englaro W., Bahadoran P., Bertolotto C. (1999). Tumor necrosis factor alpha-mediated inhibition of melanogenesis is dependent on nuclear factor kappa B activation. *Oncogene*.

[4] Xia F., Wang C., Jin Y. (2014). Luteolin protects HUVECs from TNF-*α*-induced oxidative stress and inflammation via its effects on the Nox4/ROS-NF-*κ*B and MAPK pathways. *Journal of Atherosclerosis and Thrombosis*.

[5] Liu P., Woda M., Ennis F. A., Libraty D. H. (2009). Dengue virus infection differentially regulates endothelial barrier function over time through type I interferon effects. *The Journal of Infectious Diseases*.

[6] Miyazaki K., Hashimoto K., Sato M. (2017). Establishment of a method for evaluating endothelial cell injury by TNF-*α* in vitro for clarifying the pathophysiology of virus-associated acute encephalopathy. *Pediatric Research*.

[7] Nagamine K., Furue M., Fukui A., Matsuda A., Hori T., Asashima M. (2007). Blood cell and vessel formation following transplantation of activin-treated explants in Xenopus. *Biological & Pharmaceutical Bulletin*.

[8] Nagamine K., Matsuda A., Asashima M., Hori T. (2008). XRASGRP2 expression during early development of Xenopus embryos. *Biochemical and Biophysical Research Communications*.

[9] Takino J., Nagamine K., Hori T. (2013). Ras guanyl nucleotide releasing protein 2 affects cell viability and cell-matrix adhesion in ECV304 endothelial cells. *Cell Adhesion & Migration*.

[10] Caloca M. J., Zugaza J. L., Vicente-Manzanares M., Sánchez-Madrid F., Bustelo X. R. (2004). F-actin-dependent Translocation of the Rap1 GDP/GTP Exchange Factor RasGRP2. *The Journal of Biological Chemistry*.

[11] Nagamine K., Matsuda A., Hori T. (2010). Identification of the gene regulatory region in human rasgrp2 gene in vascular endothelial cells. *Biological & Pharmaceutical Bulletin*.

[12] Stone J. C. (2011). Regulation and function of the RasGRP family of ras activators in blood cells. *Genes & Cancer*.

[13] Canault M., Ghalloussi D., Grosdidier C. (2014). Human CalDAG-GEFI gene (RASGRP2) mutation affects platelet function and causes severe bleeding. *The Journal of Experimental Medicine*.

[14] Carbo C., Duerschmied D., Goerge T. (2010). Integrin-independent role of CalDAG-GEFI in neutrophil chemotaxis. *Journal of Leukocyte Biology*.

[15] Stadtmann A., Brinkhaus L., Mueller H. (2011). Rap1a activation by CalDAG-GEFI and p38 MAPK is involved in E-selectin-dependent slow leukocyte rolling. *European Journal of Immunology*.

[16] Katagiri K., Shimonaka M., Kinashi T. (2004). Rap1-mediated Lymphocyte Function-associated Antigen-1 Activation by the T Cell Antigen Receptor Is Dependent on Phospholipase C-*γ*1. *The Journal of Biological Chemistry*.

[17] Dupuy A. J., Morgan K., Von Lintig F. C. (2001). Activation of the Rap1 Guanine Nucleotide Exchange Gene, CalDAG-GEF I, in BXH-2 Murine Myeloid Leukemia. *The Journal of Biological Chemistry*.

[18] Riches J. C., O'Donovan C. J., Kingdon S. J. (2014). Trisomy 12 chronic lymphocytic leukemia cells exhibit upregulation of integrin signaling that is modulated by NOTCH1 mutations. *Blood*.

[19] Stork P. J. S., Dillon T. J. (2005). Multiple roles of Rap1 in hematopoietic cells: Complementary versus antagonistic functions. *Blood*.

[20] Cullere X., Shaw S. K., Andersson L., Hirahashi J., Luscinskas F. W., Mayadas T. N. (2005). Regulation of vascular endothelial barrier function by Epac, a cAMP-activated exchange factor for Rap GTPase. *Blood*.

[21] Birukova A. A., Tian X., Tian Y., Higginbotham K., Birukov K. G. (2013). Rap-afadin axis in control of Rho signaling and endothelial barrier recovery. *Molecular Biology of the Cell (MBoC)*.

[22] Deng B., Xie S., Wang J., Xia Z., Nie R. (2012). Inhibition of protein kinase C *β* 2 prevents tumor necrosis factor-*α*-induced apoptosis and oxidative stress in endothelial cells: The role of NADPH oxidase subunits. *Journal of Vascular Research*.

[23] Ago T., Kitazono T., Ooboshi H. (2004). Nox4 as the major catalytic component of an endothelial NAD(P)H oxidase. *Circulation*.

[24] Basuroy S., Bhattacharya S., Leffler C. W., Parfenova H. (2009). Nox4 NADPH oxidase mediates oxidative stress and apoptosis caused by TNF-*α* in cerebral vascular endothelial cells. *American Journal of Physiology-Cell Physiology*.

[25] Wang H., Wittchen E., Hartnett M. E. (2012). The small GTPase Rap1 regulates intracellular ROS generation in RPE. *Investigative Ophthalmology & Visual Science*.

[26] Datla S. R., Peshavariya H., Dusting G. J., Mahadev K., Goldstein B. J., Jiang F. (2007). Important role of Nox4 type NADPH oxidase in angiogenic responses in human microvascular endothelial cells in vitro. *Arteriosclerosis, Thrombosis, and Vascular Biology*.

[27] Subramanian H., Zahedi R. P., Sickmann A., Walter U., Gambaryan S. (2013). Phosphorylation of CalDAG-GEFI by protein kinase A prevents Rap1b activation. *Journal of Thrombosis and Haemostasis*.

[28] Yan F., Wang Y., Wu X. (2014). Nox4 and redox signaling mediate TGF-ß-induced endothelial cell apoptosis and phenotypic switch. *Cell Death & Disease*.

